# Self‐Experienced Difficulties in Communicative Participation in Children With Speech, Language and Communication Needs—A Concept Elicitation Study

**DOI:** 10.1111/1460-6984.70170

**Published:** 2025-12-11

**Authors:** Eline Alons, Bianca Berndsen‐van Swaaij, Caroline Terwee, Ellen Gerrits, Margreet Luinge, Lizet van Ewijk

**Affiliations:** ^1^ Research Centre Healthy and Sustainable Living HU University of Applied Sciences Utrecht Utrecht the Netherlands; ^2^ Department of Languages, Literature and Communication, Institute for Language Sciences (ILS) Utrecht University Utrecht the Netherlands; ^3^ Logopedie Lentemorgen Zevenaar the Netherlands; ^4^ Epidemiology and Data Science Amsterdam UMC Location Vrije Universiteit Amsterdam the Netherlands; ^5^ Research Centre Healthy Ageing; Youth, Education and Society Hanze University of Applied Sciences Groningen Groningen the Netherlands; ^6^ Amsterdam Public Health Research Institute, Methodology Amsterdam the Netherlands

**Keywords:** children, communicative participation, patient‐reported outcome measure, speech, language and communication needs

## Abstract

**Background:**

Communicative participation is the most important outcome of speech and language therapy. A patient‐reported outcome measure (PROM) for children would help capture this outcome. Before this PROM can be developed, it is important to find out what situations children themselves find difficult because of their communication problem.

**Aims:**

The aim of the study was to identify relevant aspects of self‐reported communicative participation in children with communication disorders.

**Method and Procedures:**

Thirteen children (5–12 years old) with speech disorders, developmental language disorders (DLDs), voice disorders and/or hearing loss were interviewed with semi‐structured interviews. Before the interview they kept a diary for 1 week, documenting participation situations that were difficult because of their communication problem. Within 1 week after completing the diaries, the children were interviewed. In addition, children's ability to recall situations and reflect upon communicative participation was observed. The data analysis was conducted using directed content analysis, drawing on an existing theoretical framework.

**Outcomes and Results:**

A total of 171 situations were discussed, leading to the identification of 44 concepts, categorized into the following six categories: *person, topic, pace, location, moment* and *mode*. Some of the participants had difficulty recalling situations, and reflecting upon communicative participation. This was particularly true for children under 8 years of age (all with DLD) and two children over 8 years of age with DLD and an indication for a school for children with special needs.

**Conclusions and Implications:**

The 44 concepts provide insight into the difficulties in communicative participation experienced by children themselves. These concepts will be used to develop a PROM to assess children's communicative participation.

**WHAT THIS PAPER ADDS:**

*What is already known on this subject*

- Communicative participation is the key outcome of speech and language therapy. However, there is a lack of measurement instruments (preferably patient‐reported outcome measures, PROMs) to assess communicative participation of children. Additionally, children's own perspectives on their communicative participation, which could inform the development of such an instrument, have not yet been explored.

*What this paper adds to existing knowledge*

- This study focuses on communicative participation situations as described by children with speech, language and communication needs (SLCN). Based on children's own experiences, 44 concepts describing communicative participation were identified.

*What are the potential or actual clinical implications of this work?*

- This study enhances a comprehensive understanding of communicative participation from the perspective of children. The identified concepts can already be used in conversations with children about their communicative participation. Additionally, the findings will contribute to the development of an item bank for measuring communicative participation in children with speech, language and communication needs.

## 1 Introduction

In accessing speech and language therapy, it is essential that the voices of children and young people with communication difficulties are listened to, prioritized and acted upon (Lyons et al. 2022). Their perspectives often differ from those of adults, meaning that reliance solely on the views of clinicians or parents is insufficient (Merrick & Roulstone, 2011). Even though it is demonstrated that children with communication difficulties have the ability to reflect upon relevant factors in their lives (Markham et al. 2009), it is still argued that children's voices are often not listened to or acted upon in both research and clinical practice (Lyons et al. 2022). It is relevant for speech and language therapists to explore and elicit the child's perspectives when making decisions about speech and language therapy goals and approaches (Merrick & Roulstone, 2011). These explorations should focus on what is important to the child; the impact of the communication difficulties on all aspects of life, such as participation in school, interaction with friends and family and engagement in hobbies (Merrick & Roulstone 2011; Markham et al. 2009; Beresford et al. 2007; Cunningham et al. 2017).

The impact of having a communication problem on daily life is referred to as *communicative participation*. Communicative participation was initially defined and operationalized for adults as ‘taking part in life situations where knowledge, information, ideas or feelings are exchanged’ (Eadie et al. 2006, 309). For children with language disorders (aged 2–8), this definition was further elaborated in a Delphi study by Singer et al. (2020). They described communicative participation as ‘understanding and being understood in a social context, by applying verbal and non‐verbal communication skills’ (p. 1801). The two definitions are highly similar, but unlike Eadie's definition, Singer's version does not include written communication, due to its lesser relevance in early childhood (Singer et al. 2020).

Communicative participation has received growing attention in the field of speech and language therapy and is considered to be the ultimate goal of therapy (Westby, 2007; Baylor and Darling‐White 2020; Cunningham et al. 2017). The construct communicative participation applies to all people and is therefore relevant for all clients of SLTs that suffer from language or communication problems, regardless of aetiology (Baylor and Darling‐White 2020; Eadie et al. 2006). This allows the use of a single, universal outcome for all clients with various communication difficulties and various ages. Nevertheless, there is a substantial challenge to be addressed in order to effectively implement this participation‐focused approach, namely, the absence of assessment tools that are specifically designed to measure communicative participation in children (Kwok et al. 2022).

Measuring communicative participation is complex because it contains unobservable and subjective components that are difficult to assess with performance‐based assessment instruments (Cohen & Hula 2020; Darling‐White 2017). It is preferable to measure communicative participation from the perspective of clients themselves, by using patient‐reported outcome measures (PROMs; Darling‐White 2017). PROMs capture perceived symptoms and (limitations in) functioning in daily life, offering valuable insights into individuals’ lived experiences and perceived challenges, which may not be captured through observational or clinician‐reported tools (Yorkston and Baylor 2019; Francis 2014). However, they also have limitations, such as reliance on the respondent's cognitive and linguistic abilities, which can be particularly challenging in young children (Gale et al. 2025).

Currently, PROMs that capture communicative participation from the child's own perspective are lacking (Darling‐White 2017, Alons et al. 2024). Assessment tools that are available are either designed for the adult population (e.g., Communicative Participation Item Bank; Baylor et al. 2013; MyCommunication‐Adults; Ter Wal et al. 2024) or are reported from the perspective of parents and clinicians (e.g., Focus on the Outcomes of Communication Under Six; Thomas‐Stonell et al. 2013). Research has shown that agreement between child and proxy reports is often low, particularly for subjective and unobservable outcomes (Kwon et al. 2022). Since communicative participation includes both observable and unobservable aspects, it is essential to develop a PROM that captures children's own perspectives on their communicative participation.

In order to establish good content validity for a measurement instrument on communicative participation, it is necessary to obtain a comprehensive overview of children's perspectives on the impact of their communication difficulties on everyday participation. However, there is a paucity of research examining the impact of communication problems on everyday lives from the perspective of children themselves (Lyons et al. 2022). Specifically, there are two knowledge gaps in the current literature.

First, existing research on communicative participation in children often relies on adult perspectives. Singer et al. explored contextual factors that influence communicative participation in children with developmental language disorder (DLD) through both a systematic scoping review (Singer et al. 2022) and a qualitative focus group study with SLTs (Singer et al. 2024). The scoping review identified a limited set of personal and environmental factors, such as age, gender, being prosocial and access to therapy, that are associated with communicative participation. However, it also revealed significant knowledge gaps, particularly regarding the role of motivation, emotional functioning and interpersonal relationships. The follow‐up qualitative study provided additional insights from clinical practice, highlighting the importance of child well‐being and child‐centred SLT service provision. While these studies offer valuable contributions, they are based on adult‐reported outcomes. The perspectives of children themselves are notably absent. The studies underscore the need to further explore communicative participation from the child's own perspective, which remains underrepresented in current research.

Second, some studies did address the perspectives of children with communication problems, but they often focused on constructs related to, yet distinct from, communicative participation. For example, Markham et al. (2009) conducted a qualitative study on quality of life from the perspectives of children with various communication problems between 6 and 18 years old. The authors describe important elements of the construct quality of life in children and young people with communication difficulties, relevant to communicative participation. However, the construct quality of life is broader than communicative participation, and therefore some identified themes were related, but did not directly address communicative participation. For example, they describe themes and codes as achievement, frustration and bullying, which could be consequences of (un)successful communicative participation. Other research that includes perspectives of children themselves focused on narrow aspects of communicative participation. For example, in the work of Owen et al. (2004), children between 6 and 11 years old were interviewed about their experiences of speech and language therapy and about their general communication at school and at home. Although communicative participation will occur in these two contexts, it is likely that other contexts will also be of importance, including activities such as sports, playing outside and interacting with family members. Similarly, McCormack et al. (2010) explored how young children with speech sound disorders conceptualize ‘talking’ through drawings and accompanying interviews. While the study provides rich insights into children's experiences with talking (e.g., who they talk to, how they feel about it and what they talk about), the focus remained on the act and experience of talking itself. Communicative participation was not explicitly addressed. More research is needed to get a comprehensive overview of communicative participation from the child's own perspective.

What we do know from research in other populations provides a valuable foundation for studying communicative participation in children. Ter Wal et al. (2023) studied communicative participation in adults and identified 44 concepts affecting this area. These were further categorized into the following six themes: person, topic, location, pace, mode and moment. These categories reflect concepts that are directly linked to communicative participation as well as personal and environmental factors that influence communicative participation. Alons et al. (2025) extended this work by conducting a concept elicitation study with adolescents and young adults, identifying 37 concepts that mapped onto the same six categories. Together, they form a framework that was empirically derived from adult perspectives and has since been shown to be relevant for adolescents and young adults (Alons et al. 2025). The framework captures both generic and age‐specific aspects of communicative participation. The framework offers a promising basis for understanding communicative participation across different age groups and communication disorders and was therefore selected as the foundation for the current study. By applying this framework to children, we aim to contribute to a coherent understanding of communicative participation across the lifespan.

Research involving children's reflection on their own health‐related concepts presents age‐related challenges. Some authors suggest that children, given the appropriate tools, can reflect on health‐related constructs from the age of 5 (Varni et al. 2007), while others argue that these skills develop between the ages of 5 and 8 (Rebok et al. 2001). PROMs rely on patient self‐reporting and therefore on the respondent's linguistic and cognitive abilities, which can be particularly challenging in young children (Gale et al. 2025). Among children with communication difficulties, these age thresholds may vary due to differences in language development. Consequently, it remains unclear at what age children can effectively reflect on their communicative participation. These issues could be addressed by using communication‐supportive methods when developing new PROMs, in which simplified language and visual aids are used (Owen et al. 2004). This would allow us to explore how children of different ages and with different communicative abilities engage in PROM development research and to identify potential developmental patterns in self‐reflection.

There is a clear need for a PROM to assess communicative participation in children with communication difficulties. To develop such a measure, it is essential to explore children's perspectives on communicative participation challenges and assess their ability to articulate their experiences. Therefore, the primary aim of this study is to identify key aspects of self‐reported communicative participation in children (Ages 5–12) with communication disorders. A secondary aim is to explore how children reflect upon their communicative participation.

## 2 Method

This study involved a qualitative research design using semi‐structured interviews combined with a sensitizing exercise using diaries. Data collection was carried out by B.B. (experienced SLT and master's student) between December 2023 and April 2024, supervised by E.A. (SLT and PhD student) and L.E. (senior researcher and SLT). As an experienced SLT, B.B. was familiar with communicating with children with communication difficulties and was able to facilitate communication whenever necessary.

### 2.1 Participants and Recruitment

A purposive sampling strategy was used to recruit children between 5 and 12 years old with speech disorders, language disorders, voice disorders and/or hearing loss. Where some studies note that children from the age of 5 years old are able to provide reliable health‐related information through self‐report (Coombes et al. 2021; Varni et al. 2007), others mention that this is possible from the age of 8 years old (Matza et al. 2013). In this study, it was decided to include children from the age of 5, and to observe their ability to reflect upon communicative participation in a conversation.

Participants were recruited in three ways as follows: (1) through messages via the Dutch Association of Speech and Language Therapy (NVLF); (2) social media (LinkedIn, Facebook pages for SLTs or parents); (3) network of the researcher B.B.

The following sociodemographic variables were collected: age, gender, diagnosis (as mentioned by the parents of the participant), type of education (regular vs. special education) and school year (i.e., the year or group the child was in at primary school). In the Netherlands, *regular education* refers to mainstream schools. *Special education* is intended for students with more significant learning, behavioural or physical challenges. There are dedicated special education schools that offer adapted teaching methods, smaller class sizes and specialized staff to meet the needs of their students.

The aim was to include at least 12 participants, representing all sub‐populations, considering the type of disorder (speech, language, voice, hearing). Recruiting participants continued until no new key concepts were identified.

### 2.2 Ethical Considerations

This study was approved by the Internal Review Board of HU University of Applied Sciences Utrecht (reference number 211‐000‐2022) and was conducted according to the principles of the Declaration of Helsinki (World Medical Association 2013) and in accordance with the Dutch Medical Research Involving Human Subjects Act (WMO).

Parents were provided with an information letter about the study. This letter contained a QR code to an information video for children. Parents were asked to watch and discuss this video with their child. Prior to signing informed consent by the parents, the researcher (B.B.) hosted a short introductory meeting to answer questions of the participant and parents.

As a token of appreciation, children received a small gift (e.g., a tic‐tac‐toe game) after completing the interview. No financial compensation was provided.

### 2.3 Data Collection

This study involved in‐depth interviews about self‐experienced communicative participation using diaries as a sensitizing exercise.

#### 2.3.1 Diaries

Children were asked to keep a daily diary during 1 week in which they reported on participation situations that they experienced as difficult because of their communication problems. The diary was provided in a standard format (Appendix 1), and children were allowed to keep the diary in their own preferred way (e.g., drawing, writing, making photos, etc.). The diary included an explanation in simplified, age‐appropriate language (e.g., participation situation was explained as the moments when you were unable to take part). In addition, the researcher explicitly discussed possible difficult words and the purpose of the diary during the introductory meeting, to ensure that everything was clearly understood by the participants.

The diary was pilot‐tested with five children aged 6–10 years with speech and/or language disorders, some attending special education. Informal feedback showed that one of the original questions, ‘How did it end?’ often led to vague answers like ‘good’. Replacing it with ‘How did you solve it?’ improved the detail and relevance of responses. In addition, children with more complex communication needs benefited from explanations during an introductory session and follow‐up contact with parents and the child a few days after starting the diary to address any questions or difficulties.

Participants received reminders and could contact researcher B.B. for assistance or questions while completing the diary. Parents were allowed to help their children if needed, but were given clear instructions emphasizing the importance of capturing the child's perspective. This approach minimized parental influence on the content. Once completed, the diaries were submitted to the interviewer. They were used to generate sensitizing concepts that informed the development of the interview topic list.

The diaries were used to facilitate the recall of situations. Young children have difficulty recalling over a longer time period, and have difficulty comprehending the concept of time (Arbuckle & Abetz‐Webb 2013; Matza et al. 2013; Rebok et al. 2001). Recalling specific, concrete events is easier to recall than recalling a time frame. The use of diaries enabled the interviewer to ask the children to reflect upon concrete events (e.g., *‘I see that you were playing football at school during the break, and then there was an argument between you and XX. Can you tell more about that?’*), instead of asking to reflect upon a time frame (e.g., *‘Can you tell what happened in the schoolyard on Tuesday?’*), and thereby supported the recall process.

A further advantage of using diaries was that the interviews focused on contexts the children themselves identified as meaningful. This ensured that the discussions reflected the child's perspective rather than the researcher's preconceptions. By using diaries as a sensitizing tool, the interview guide was tailored to include topics chosen by the children, resulting in more child‐centred and relevant interviews.

#### 2.3.2 Interviews

Within 1 week after completing the diaries, children were interviewed. Interviews took place either in person or (when preferred by participant) online via Microsoft Teams and were conducted in Dutch. The interview guide was based on the concept elicitation guidelines of Patrick et al. (2011). These guidelines provide a structured approach for identifying relevant concepts from the target population's perspective, particularly in the development of PROMs. This was further supplemented by the topics covered in the diary (Appendix 2).

Parents of the children were allowed to be present during the interview, but were asked not to interfere with the conversation. If necessary, the interviewer asked to assist in recalling the situation from the diary based on their own knowledge (e.g., ‘*Remember, it was on the day we went to grandma and afterwards to the supermarket*.’).

The diary was on the table during the interview and was used to look at together. To maintain engagement, the interviewer used several child‐friendly strategies, such as visual aids (e.g., drawings), playful questioning and interactive elements (e.g., pointing at diary entries together). Additionally, having a familiar toy on hand, taking drink breaks in between, engaging interactively by drawing and writing together and allowing the child to show their own materials helped create a comfortable and engaging atmosphere. If necessary, communication during the interview was supported by the interviewer, using drawings and written support (Barlow et al. 2011; Holliday et al. 2009; Brooks 2005). During the interviews, the researcher often used summarizing questions to ensure that the child's intended meaning was correctly understood.

### 2.4 Data Analysis

All interviews were transcribed verbatim. Analysis was conducted by B.B. and E.A., using the software ATLAS.ti (Atlas.ti 2022). Discrepancies were discussed until consensus was reached.

#### 2.4.1 Diary Analysis

The diaries served as a sensitizing tool in preparation for the interviews and were not systematically coded or analysed as standalone data sources. Prior to each interview, the researcher reviewed the diary to identify specific situations described by the child. The situations formed the basis for personalized interview questions, as reflected in the interview guide (Appendix 2, under the heading ‘diary content’). In this way, the content of the diaries was integrated into the interview process, without conducting a formal content analysis of the diaries themselves. This part of the analysis was performed by B.B.

#### 2.4.2 Interview Analysis

We aimed to build upon the framework proposed by Ter Wal et al. (2023), which provides a structured approach to understanding communicative participation based on interviews with adults. Previous research has shown this framework also applies to adolescents and young adults (Alons et al. 2025). Given that communicative participation is considered a generic construct, relevant across age groups and types of communication disorders (Baylor and Darling‐White 2020), our goal was to contribute to a coherent understanding of this construct across the lifespan. To achieve this, we employed an analysis strategy that allowed for both deductive and inductive reasoning. We therefore conducted a directed content analysis (Hsieh and Shannon 2005), which is particularly suited for studies that seek to validate or extend an existing theoretical framework. The pre‐existing categories, as described by Ter Wal et al. (2023), were used as a foundation and were further refined and expanded based on the findings of the present study.

The analysis process consisted of several iterative steps. First, all transcripts were read carefully to allow the researchers to familiarize themselves with the data and gain an overall understanding of each participant's experiences. In the next step, relevant fragments were identified—specifically, segments describing life situations in which participants experienced difficulties communicating. These fragments were then coded deductively, using the pre‐existing codes from the framework developed by Ter Wal et al. (2023). When fragments did not align with any of the existing codes, new codes were developed inductively based on the content of the data. Throughout the analysis, a process of constant comparison was applied, in which codes were repeatedly compared across transcripts and refined as needed to ensure consistency and analytical depth. Finally, all codes were organized into one of the six overarching categories defined in the original framework by Ter Wal et al. (2023), with minor adjustments made where appropriate to accommodate new insights.

The analysis of the interviews was conducted by the authors (B.B. and E.A.). They both coded the transcripts and then discussed their findings. Differences were discussed until a consensus was reached.

### 2.5 Observations Recall and Reflection on Communicative Participation

As it is unclear at what age children can accurately reflect upon a complex construct as communicative participation, we decided to include children as young as 5 years old and to observe their ability to participate in an interview on their self‐experienced communicative participation. This observation was performed by the interviewer, who subjectively assessed the child's ability on two components as follows: ability to recall situations and ability to reflect upon communicative participation. These components were assessed using the following four criteria:− *= difficult, much help needed; ±− = fair possible, much help needed; ± = fair possible, with help if needed; + = possible, with help if needed*.

## 3 Results

Thirteen children participated in the study, of which nine were boys and four were girls, with DLDs (*n* = 10), speech disorders (*n* = 6), hearing loss (*n* = 2), voice disorders (*n* = 1) or a combination of them (*n* = 5). The age of the children varied from 5 and 11 years old. The mean age of the participants was 8.3 years. Eleven children attended regular elementary school (RES), two of them with an indication for special needs elementary school (SNES). Two children attended SNES. Nine children were monolingual, four were multilingual. The characteristics are presented in Table 1. All participants participated verbally in the interview.

**TABLE 1:** Baseline characteristics participants.

Participant	Gender (M/F)	Age (years;months)	Communication disorder^a^	Level of education (form, school year)
1	M	6;04	Language, speech	RES, 2
2	M	6;08	Language	RES, 3
3	M	5;00	Language, speech	RES, 1
4	M	10;06	Language	RES^b^, 6
5	M	11;01	Language	RES, 7
6	M	9;02	Language	RES, 5
7	F	9;09	Language	RES, 6
8	M	5;07	Language, speech	RES, 2
9	M	8;09	Speech	SNES, 5
10	F	8;04	Language, speech, hearing	RES^b^, 4
11	M	7;05	Language, hearing	SNES, 4
12	F	10;05	Speech	RES, 7
13	M	8;05	Voice	RES, 5

*Notes*: M = male, F = female. Language = developmental language disorder (DLD); speech = stuttering, phonological disorder, general speech disorder or related to cleft palate syndrome; hearing = one‐sided deafness or hearing loss; voice = hoarseness.

Abbreviations: RES, regular elementary school; SNES, special needs elementary school.

^a^ Disorder as reported by the parents/caregivers.

^b^ Transfer to SNES upcoming.

In total, 171 situations were discussed with the children. Situations reflected a variety of participation situations. Based on all the discussed situations, 44 concepts were identified. The concepts are presented in Table 2. The categories and their related concepts are described below.

**TABLE 2:** Identified concepts.

	Person	Topic	Pace	Location	Moment	Mode
**What concepts describe communicative** **participation in children with communication disorders?**	Friends	Humour	Fast changes in communication	School	Emotions	(Video)calling
	Family	Solving problems	Speaking rate	Playground/Outside	Fatigue	Presenting
	Parents/Caregivers	Arguments	Waiting for turn causes forgetting	Sports	Insecurity	WhatsApp
	School teacher	Expressing feelings and wishes		Party	Nervous	Reading as a prerequisite for learning
	Health care professional	Play		After‐school care		
	Siblings	Consulting		Hair salon		
	Childcare worker	Instructions		Store/Market		
	Classmates	Questions		Talking distance		
	Sports teacher	Time aspects		Surroundings		
	Other children (peers, older, younger children)	Linguistic complexity				
	Unfamiliar people	Conversation topic				
	Multilinguism					
	People with hearing problems					

The interviews lasted 35–60 min. The quotations presented in this article have been translated into English. In the translation, we tried to maintain the features of the original formulations by not converting the utterances into written language conventions. When utterances did not meet the standards of spoken Dutch, we tried to transfer these characteristics to the English translation.

### 3.1 Person

One factor that plays an important role in communicative participation is the conversational partner. This was mentioned by participants with all types of communication problems. Two kinds of difficulties were experienced by the children.

First, children indicated that the social role people have influences their level of difficulty with communicative participation. Children identified different social roles of people with whom they needed to talk, including *friends, family, parents/caregivers, school teacher, health care professional, siblings, childcare worker, classmates, sports teacher, other children* and *unfamiliar people*. Children experienced more problems with some roles than others. For example, talking to a stranger was considered difficult, as presented in the following quote:

*P7: Uhm, one time I asked so, my mother sent me to the supermarket for some baking powder, or yes baking powder. And my mother said that color it has to be and that and that and this bi. . and it has to be this big. And there were no more big ones. I also asked the cashier and also people in the shop, who couldn't find it either. The cashier said: they don't have big ones anymore, they're out of them. And uh so I took the small one. Uh, and after that, because at first uh she didn't understand me that I wanted the big one. When she understands I mean those big baking powders, but they were out. (girl, 9 years old, language problem)*

Second, children described specific characteristics of the conversation partner that influenced their difficulty with participation, which were not linked to a social role. One example was multilingualism, where multilingual children found it more difficult to engage with people who spoke a different language. Another frequently mentioned characteristic was hearing impairment.

Hearing difficulties were mentioned in four situations as follows: three involving grandparents and one involving a healthcare professional. These examples came from children who themselves experienced challenges with speech or voice production. In such cases, the participation restriction appears to stem from both sides of the interaction. As one child explained:

*P12: Grandma was on the phone. […] Yes, grandma didn't hear me, because, yes she has, she didn't have the hearing aid in then I think. So yeah, she cannot hear very well. (girl, 10 years old, speech problem)*

Additional context was provided by a parent, describing a situation with a doctor:

*M12: ‘And the doctor herself said that she didn't hear very well. That also plays a role. Then it's a double challenge. There's a communication problem on both sides, which makes communicative participation difficult.’ (mother of girl, 10 years old, speech problem)*

These examples illustrate that communicative participation is not solely dependent on the child's abilities, but also on the characteristics of the conversation partner. When both parties experience communication difficulties, the interaction becomes significantly more challenging.

### 3.2 Topic

Children talked about situations where the topic of the conversation affected how well they participated. Three types of the topic can be distinguished. First, children mentioned topics with emotional weight, like *humor, solving problems, arguments* and *expressing feelings and wishes*.

*[P8 Talking about problem with little brother during play]*

*BB: Hey, and when that happened to [little brother], huh, could you tell [little brother] you didn't like it?*

*P8: No, I, I, I was rea. . rea. . really sad, because he I. . Because I like it só much*.

*BB: Oh yes. But were you super angry with your little brother then?*

P8: Yes.

*Yes. And were you able to solve that with words or not?*

*P8: No uh he, un, un, didn't understand uh aaaanything. (boy, 5 years old, language and speech problems)*

*P7: Sometimes I don't get the joke and then they kind of have to explain it. Because I remember once, uh uh then I went to a party of a friend of mine at gymnastics and there we went ice skating. And then I didn't understand, because she had a kind of a little note. Uh, because she gave us kind of a treat at the end with a note like that and it said: hope you liked my cool party. And then, I didn't understand. So I had… until I asked the other girls. They said: don't you understand, cool party, from cold, kind of, yeah. (girl, 9 years old, language problem)*

Second, participants mentioned that they experienced difficulties participating in situations that required consulting, described by the concepts of *play* and *consulting*. They talked about finding it difficult to communicate with other children about the rules or plans during play or when consulting with each other in other situations than play (e.g., making homework).

*Diary situation: Consulting with a friend about what to play, when television is on and adults are talking with each other*.

*P12: The TV was on, so then we couldn't really understand each other very well. And mum was also talking to her parents then. So that's why. *.

*BB: So then there was a lot of sound at the same time actually: and from the TV, and from talking*.

*P12: Yes*.

*BB: So then she couldn't understand you?*

*P12: Yes. (girl, 10 years old, speech problem)*

Last, participants mentioned many situations that were difficult because of the complexity of the message. This is described by the concepts of *questions, instructions, linguistic complexity, time aspects and conversation topic*. Understanding an instruction, asking a question, and talking about a topic that contains time (e.g., future or past events) appeared to be complex topics which are more difficult to talk about. In the following quote, P7 with DLD reflects upon a situation during gymnastics in which she finds it difficult to understand the teacher. She also reflects upon solutions that help her understand the teachers’ explanations.

*P7: Yes. And then uh we just go for example balance beam. Then the teacher. . We go and stand on the beam. The teacher stands on the floor. Then she shows us what to do. And then she explains it. And she explains how you do that*.

*BB: So she explains it, but she shows it at the same time, you mean?*

*P7: Yeah*.

*BB: And is that easier for you then, because you can see it?*

*P7: Yes. Yes, because if I can also see a picture of something, then I also understand more what it means*.

*BB: Yes, okay. But in gymnastics, do you ever have the teacher tell you something without you being able to see it?*

*P7: Uh, yes, she says that sometimes, but she, sometimes she says: there you do this and then you do this and then this. But if you don't understand, the teacher really always, always asks: are there any questions? I also had that when I just started. I didn't understand how I, how… […] I didn't understand what things were, but now I do. Because in one… because in that one day, I had raised by finger on almost everything. (girl, 9 years old, language problem)*

For many participants, it was difficult to communicate about topics that involved time aspects. Both understanding time aspects and telling about past and future events were difficult. This concept was only mentioned by the participants with language difficulties.

*[interviewer reading information from diary]: So on day 5 you were in [name city] and then you couldn't buy the chips. But the day after, your sister had bought the chips. You didn't know she was going to buy it?*

*P6: No I didn't know that. I didn't know anything about it. She did say that, I'm going to get your chips. But I don't know… she said tomorrow, but I thought uh… She meant, I just thought she meant, meant, the day after tomorrow*.

*BB: Aah, so your sister had told when she was going to get it, but you didn't understand correctly?*

*P6: Yes. (boy, 9 years old, language problem)*

### 3.3 Pace

Children mentioned that the pace of the communication plays a role in their communicative participation. This was reported by participants across all types of communication problems. Conversations with both a fast pace and a slow pace were difficult. The following concepts were identified: *fast changes in communication, speaking rate* and *waiting for turn causes forgetting*. An example of *waiting for turn causes forgetting* is described by the following quote:

*P1: When I, when uh, when uh, uh mum is talking to dad. And uh, I say ‘mama’, he goes on talking to daddy, I forget something!*

*BB: Ooh, so if you have to wait a while before you can say something, then you forget again*.

*P1: Yes! (boy, 6 years old, language and speech problems)*

### 3.4 Location

The location where communication takes place plays an important role in the communicative participation. This category can be divided into two groups. First, children mentioned specific places where they often have trouble communicating. These places include school, playgrounds, sports activities, parties, after‐school care, hair salons and stores. Second, participants mentioned environmental factors that influences the communicative participation. These factors are not linked to a specific location. This includes the concepts *surroundings* and the *talking distance*, that were often mentioned by people with speech, voice or hearing problems. Children described that they have more difficulties participating in conversations in noisy or crowded places.

*BB: At a party sometimes there is quite a bit of noise, how is that?*

*P13: That does bother me sometimes. […] At my best friend's children's party. Then we had a sleepover party. So then we were playing really loud music upstairs. I wanted to say: ‘can you turn it down a bit? But then they didn't hear me. (boy, 8 years old, voice problem)*

The concept *talking distance* was mentioned by two participants, one with speech problems and one with voice problems. A greater distance seems to increase the chances of problems in the communication.

*BB: Yes, or is there any other time at all, that mum can't hear you properly? [. .]*

*P12: Uh, when mum is upstairs and I'm downstairs. Or vice versa. That's a bit long distance. Upstairs to downstairs. (girl, 10 years old, speech problem)*

### 3.5 Moment

Successful communicative participation depends on the moment in which communication takes place. This was mentioned by children with all types of communication problems. Concepts that were identified within this category are *emotions* (feeling sad or angry during a conversation), *fatigue, insecurity* and *nervous*. How children feel impacts them and has influence on their communicative participation.

*P9: [conversation about talking to people you don't know]. Yes, because, because then I get a bit vernous (nervous). That later someone, that later then do things that… because they think … that I, that, then I'm going to get vernous, for no reason, because I don't know then what to do. (boy, 8 years old, speech problem)*

### 3.6 Mode

The mode of communication plays an important role in communicative participation. Concepts within this category were mentioned by children with language or speech disorders only. Different modes were described that were experienced as being difficult. For starters, the concept *presenting* was mentioned, which refers to giving a presentation in class. This often comes with nervousness and insecurity.

*P4: At a presentation of my story in grade 6… Yes, then I had to talk about a sports club and it was about Paris. Uhm, uh, and uh, yes, I don't actually remember, because I even forget a lot… But, yes, then I had even said a few words wrong and still, I was way too nervous, I was pacing back and forth all the time. And yes, and almost didn't look at the class at all. (boy, 10 years old, language problem)*

Other concepts that relate to the category mode were *(video)calling*. There were two concepts identified that contained written language: *WhatsApp* and *reading as prerequisite for learning*. These concepts were particularly mentioned by the participants with language problems. In reading, participants mentioned problems when reading together with parents, as well as participating in reading comprehension in class.

### 3.7 How Children Reflect Upon Their Communicative Participation

Some children had difficulty recalling situations and reflecting upon communicative participation. This was particularly true of the children under 8 years of age (all with DLD) and two children over 8 years of age with DLD and an indication for a school for children with special needs. The subjective overview of the observations is presented in Table 3.

**TABLE 3:** Observations in ability to recall situations and reflect upon communicative participation.

Participant	Recalling situations	Reflecting upon communicative participation
1 (6.33; RES, 2; language, speech)	±	−
2 (6.08; RES, 3; language)	±	−
3 (5.00; RES, 1; language, speech)	−	−
4 (10.50; RES^a^, 6; language)	±	±
5 (11.08; RES, 7; language)	+	+
6 (9.17; RES, 5; language)	+	+
7 (9.75; RES, 6; language)	+	+
8 (5.58; RES, 2; language, speech)	±−	−
9 (8.75; SNES, 5; speech)	+	+
10 (8.33; RES^a^, 4; language, speech, hearing)	±−	−
11 (7.42; SNES, 4; language, hearing)	±−	−
12 (10.42; RES, 7; speech)	+	+
13 (8.42; RES, 5; voice)	+	+

*Note*: participant = participant number; age; educational level; disorder. − = Difficult, much help needed; +/− = fair possible, much help needed; +/− = fair possible, with help if needed; + = possible, with help if needed.

^a^ Transfer to SNES upcoming.

## 4 Discussion

This study aimed to identify relevant, self‐reported aspects of communicative participation in children (5–12 years) with speech, language, voice disorders and/or hearing loss. In total, 44 concepts were identified related to the *person, topic, pace, location, moment* and *mode of* communicative participation. While previous studies have explored children's perspectives, they often focused on related but distinct constructs, such as quality of life or general communication skills, rather than communicative participation specifically. Moreover, research on communicative participation has typically centred on older age groups, such as adults, adolescents or young adults, or relied on adult proxies like parents or professionals to represent the child's experience. This study addresses these gaps by providing a structured, concept‐level overview of communicative participation based on children's own reports. These findings offer insight into the specific conditions and challenges children associate with daily communication. The implications of these gaps—both in terms of construct and target group—are discussed in more detail below.

### 4.1 Relating Children's Perspectives on Communicative Participation to Other Constructs

The results identified in this study provide insight into the perspectives of children on their own communicative participation. If we look at literature that focuses on exploring children's perspectives on (aspects of) their daily lives, several studies show similarities and differences. In an attempt to take a first step in developing a measurement instrument for assessing quality of life, Markham et al. (2009) conducted focus groups with children and adolescents aged 6–18, including a broad target group with various communication difficulties. In the work of Owen et al. (2004), interviews were conducted with children aged 6–11 who had speech and language problems. The goal was to gather experiences with speech and language therapy from the children's own perspectives. The approaches of both studies share similarities with this work, particularly in the chosen age group, in obtaining children's perspectives, and in the diversity of communicative challenges. There are also similarities in the results. For instance, Markham et al. (2009) highlights the importance of social interactions and strong support for children's well‐being. The related subthemes largely describe specific individuals with whom the child interacts socially and from whom they receive support. Owen et al. (2004) also discusses the theme of social interaction and emphasizes that children consider having friends to be extremely important. These findings strongly align with the concepts identified under the category *Person*. Other similarities can be found in the theme of *School*, which plays a significant role in both Owen's and Markham's studies. They describe the importance of a quiet and calm school environment. These elements are also present in this work but are categorized under different concepts (e.g., *location—school* and *location—surroundings*).

Differences can also be noted. An important category identified in this work is *Mode*, which describes the significant role of written communication in communicative participation. In Markham's and Owen's studies, this form of communication did not appear in the results. In contrast, Markham and Owen identified themes that did not appear in this study. For example, Markham describes themes that are broader than communicative participation (e.g., achievement, awareness and relaxation) and Owen describes perspectives on therapy. We believe that all this work complements each other and helps both research and clinical practice to understand the perspectives of children, exactly what is needed so much (Lyons et al. 2022).

### 4.2 Towards One Framework for Communicative Participation From the Perspective of Children

Most identified difficulties were related to the communication partner, emphasizing their importance for children with communication challenges. This study identified specific roles (e.g., family, friends, healthcare professionals and unfamiliar people) and general characteristics of communication partners. Compared to Ter Wal et al. (2023), who conducted a concept elicitation on communicative participation in adults, and Alons et al. (2025), who conducted a concept elicitation in adolescents and young adults, similar role distinctions were found. However, children mentioned additional roles like school teachers, caregivers, child workers and classmates. In adolescents and adults, concepts as facial expressions and non‐verbal communication were identified that were not mentioned by children. This last finding is somewhat surprising, considering the well‐established importance of non‐verbal information in early communicative development. For example, the ‘still face’ experiment (Tronick et al. 1978) illustrates how crucial facial expressions are in early parent–child interactions and in the development of communicative competence.

There are several possible explanations for why this concept did not emerge in our data. First, it may be related to limited data saturation, as our sample consisted of only 13 children. Although the interviews yielded rich insights, it is possible that certain aspects of communication, such as non‐verbal elements, were not captured due to the sample size. Second, children may not be fully aware that non‐verbal behaviours are also forms of communication. Although facial expressions are used in their communication, they are not yet able to reflect on this aspect of communication. Third, the interview context may have influenced the salience of verbal over non‐verbal aspects. The framing of questions and the verbal nature of the elicitation process may have steered children toward describing spoken language, rather than non‐verbal forms of communication.

These considerations suggest that children may implicitly use non‐verbal cues, but lack the metalinguistic awareness to articulate their communicative function. As a result, they tend to experience and describe communicative participation primarily within the framework of verbal language. This developmental gap between use and awareness has important implications for both research and practice. If non‐verbal aspects of communication are present but not verbalized by children, then self‐report methods may not fully capture their communicative participation. To obtain a more complete and accurate understanding, it may be necessary to complement children's own reports with proxy questionnaires or observational methods. This approach ensures that the full range of communication is acknowledged.

In the *Topic* category, 11 concepts emerged, related to emotional weight, those requiring consultation and messages with complexity. While previous studies identified emotional and complex topics, the consultation aspect is unique to this study. We use the term *consultation* to describe peer interactions that involve joint decision‐making, negotiation and coordination—processes that require children to express their views, listen to others and reach mutual agreements. Many children described these situations as challenging, as they involve explaining one's own perspective, listening to others, negotiating and sometimes resolving conflicts. Examples include discussing how to play a game, making plans together or working on a group assignment. These interactions require social and communicative skills that children between 5 and 12 years are still developing. Therefore, they often perceive such moments of consultation as complex and effortful. Concepts such as emergencies, politics and appointments, noted by Ter Wal et al. (2023), fit better under practical life situations than consultation.

For *Location*, a distinction was identified describing specific places where communication occurs and general characteristics like talking distance and surroundings. The more general characteristics were also identified in Ter Wal et al. (2023) and Alons et al. (2025). When it comes to concepts describing specific locations, there are differences in the identified concepts. There are some concepts (*playground/outside* and *after‐school care)* that were not identified in previous studies. In this study, we did not identify concepts as *work*. These differences represent the changes in social contexts that take place when growing up, leading to different contexts being relevant.

In *Pace, Moment and Mode*, findings aligned with previous studies, but two new concepts emerged. Within *Pace*, children described *waiting for turn causes forgetting*, a challenge not emphasized by older groups. In *Mode*, fewer concepts were identified compared to Ter Wal et al. (2023) and Alons et al. (2024), particularly regarding written communication. Adults and adolescents frequently mentioned email, personal mail and texting, while children focused on WhatsApp and reading as prerequisites for learning. These findings suggest that written communication gains importance with age.

There are many similarities between all the concept elicitation studies, from which we can conclude that communicative participation is indeed a generic construct that applies to everyone (Baylor and Darling‐White 2020). However, because communicative participation is about life situations that are different for each individual, we also see differences in the concepts identified in different age groups, especially those related to a place or a person. Capturing a complex construct such as communicative participation is complicated, but by focusing on the core concepts, we seem to be highlighting some important similarities and differences that will be important in the development of measurement tools.

### 4.3 Minimal Age for Reflection on Communicative Participation

The literature on the use of PROMs in paediatric populations varies significantly regarding age limits. Some authors suggest that children as young as five can report on aspects of their health (Coombes et al. 2021; Varni et al. 2007), while others describe exact age cut‐offs as ‘fuzzy’ (Arbuckle and Abetz‐Webb 2013). The appropriate cut‐off often depends on the population and the construct being measured, leading to the recommendation of a broad range between 6 and 8 years (Arbuckle and Abetz‐Webb 2013). Variability in cognitive and linguistic development among children further complicates these determinations (Bevans et al. 2020).

When considering communication‐related constructs, existing literature presents both similar and contrasting findings. Markham et al. (2009) recruited children as young as six for their study on quality of life in children with communication problems, basing their age cut‐off on research suggesting that children under six are unlikely to engage in group discussions (Heary and Hennessey 2002) and that the ability to reflect on health status develops between 5 and 8 years old (Rebok et al. 2001). While they reported positive participation outcomes, their study only included children with sufficient receptive and expressive language skills to engage in group discussions with minimal support. In contrast, Owen et al. (2004) found that children aged 6–11 were not always aware of their communication difficulties or why they received speech and language therapy. Similarly, McCormack et al. (2010) observed that preschool children with speech disorders did not explicitly describe their speech as a problem, although they exhibited communication difficulties during conversations, including during interviews.

For this study, we chose to include participants as young as five. However, we found that younger children (<8 years), particularly those with DLD, struggled to reflect on their communicative participation. Additionally, some older children with special educational needs faced similar challenges. These findings align with those of Owen et al. (2004) and McCormack et al. (2010), suggesting that linguistic abilities, cognitive development and the complexity of communicative participation likely contributed to these difficulties. While our observations cannot definitively determine causality, they highlight the importance of considering cognitive and linguistic factors when assessing children's ability to self‐report on communication‐related constructs.

Although it would be beneficial for SLTs in clinical practice to have an established age limit when using measurement tools and actively engaging children in discussions about their daily experiences, this has proven to be a complex challenge. The ability to reflect on communicative participation is not strictly tied to a specific age. Nevertheless, we strongly advocate for initiating conversations with children about their daily experiences whenever possible. Further research is required to develop and validate measurement tools for this purpose, and such developments are currently underway.

### 4.4 Study Limitations

It is important to acknowledge the limitations of this study, which may have influenced the interpretation of the findings. We aimed to include a diverse group of participants with various communication problems. However, we included many children with language disorders, while only two children with hearing loss were included and one child with a voice disorder. In addition, we only included children who were able to participate in the interviews verbally. We have not succeeded in including children who use augmentative and alternative communication methods. It would be of added value to obtain their perspectives as well and further complement the framework of communicative participation with these experiences.

A second limitation is that the method regarding the minimal age to reflect upon communicative participation solely relies on observations that were very exploratory. Since little information was available regarding cognitive and linguistic requirements for reflection on health and health‐related constructs, especially in a communication vulnerable population, this study provides a starting point, but more research in this population is required.

A third limitation is that although we wanted to obtain the perspectives of children, it became evident that for some children, the assistance of parents was needed, particularly with completing the diaries. However, during the interview, the child itself was asked about their own experiences within the situations mentioned in the diary. If they did not recognize the situation or did not see the situation as a problem, this soon became apparent during the interviews. By using this approach, we tried to obtain the child's perspective as much as possible. The method is, however, not foolproof, and it might be that during the diary phase, the perspectives of the parents have interfered with the perspectives of the child.

We tried to further complement the framework that was originally developed by Ter Wal et al. (2023) and further complemented by Alons et al. (2025). However, in all three studies, interviews were conducted by different interviewers and analysed by different persons. The differences in identified concepts could also be due to a (slightly) different perspective of the researchers, which is common when conducting qualitative research. A secondary analysis of all data could be of added value to eventually create one framework from one perspective.

### 4.5 Clinical Implications

These findings provide unique insights into the self‐perceived barriers children face in communicative participation. These insights are highly relevant for clinical practice. Speech and language therapists should actively work with these child‐identified concepts. By using them as conversation starters, they can engage children in shared goal setting and ensure that therapy aligns with the communicative challenges children experience in daily life. Integrating these real‐life contexts makes it possible to offer more targeted and meaningful support. This study underscores the urgency for SLTs to move beyond general language goals and address the specific communicative demands children encounter, making their interventions more effective and relevant.

### 4.6 Conclusion and Future Directions

In conclusion, we obtained a comprehensive overview of relevant, self‐experienced concepts that influence communicative participation. These concepts further complement the framework previously developed in other populations (adults; Ter Wal et al. 2023; adolescents; Alons et al. 2025). This study forms an essential step toward developing a PROM that reflects communicative participation from the child's own perspective.

The results of this study will be combined with findings from a literature review on existing PROMs to inform item generation. In the next phases, children will be actively involved in formulating items, shaping the structure of the instrument, and contributing to a content validity study. This child‐centred approach will ensure that the final PROM is comprehensible, relevant and truly aligned with the lived experiences of children.

## Funding

This study was partially supported by funding from ZonMw (reference number 10270032021301). While the data were collected by a master's student, the supervision and scientific embedding took place within a project funded by ZonMw.

## Ethics Statement

This study was approved by the Internal Review Board of the HU University of Applied Sciences Utrecht (reference number 211‐000‐2022).

## Consent

The authors confirm that written informed consent has been obtained from the parents of the recruited participants.

## Conflicts of Interest

The authors declare no conflicts of interest.

## Data Availability

The data that support the findings of this study are available from the corresponding author upon reasonable request.

## Appendix 1 Children's Diary

### 1.1

Day 1

## Appendix 2 Interview Guide

### Warm‐Up Question

- You kept a diary for this interview; what was it like for you to keep this diary?

### General Questions About Difficulties in CP

- In which situations do you find it difficult to understand what someone is saying to you? Can you describe that situation?
- In which situations do you find it difficult to tell something to someone? Can you describe that situation?
  è If a child does not come up with situations that are difficult, indicate that this will become clear when discussing the diary.

#### Diary Content

Present the diary and invite the participant to look at their diary with you. Discuss the diary day by day.

- Tell me about what you draw/wrote/photographed here.
  è Why did you write/draw/photograph this situation?
  è Additional questions to adequately understand what the child is telling.
- What was difficult in this situation?
- Why was this difficult?
- How did you react in this situation?
- What did you do to solve the problem?
- Did someone help you to solve the problem? What was helpful?
- Are there more situations like this where you experienced difficulties participating?
  è If so, in which situations?
- Can you tell me about something your good at? (*positive ending of questioning*)

Summarize and check with the participant if the answers are understood correctly.

#### Finishing

- Summarize the findings.
- Ask if the participant wants to add or revise the findings.
- Thank for participating and hand over a present for the child.
